# Endoscopic Submucosal Dissection of Cecal Lesion via Redirectable Traction Control Technique Using a Clip‐Band Traction Device

**DOI:** 10.1111/den.70278

**Published:** 2026-09-13

**Authors:** Takahiro Muramatsu, Masakatsu Fukuzawa, Takao Itoi

**Affiliations:** ^1^ Department of Gastroenterology and Hepatology Tokyo Medical University Hospital Tokyo Japan

The cecum has thin muscular layers and forms a blind end; therefore, during endoscopic submucosal dissection (ESD), the muscular layer is vertically oriented against the knife, thereby increasing the risk of perforation [1]. Additionally, the cecum is a closed space, which restricts endoscopic maneuverability. Although the pocket creation method and traction devices can be useful for cecal ESD [2, 3, 4], an optimal strategy has not been established. Notably, a recent study demonstrated the usefulness of a novel clip‐band traction device for colorectal ESD [5]. Herein, we present a video of successful ESD for a cecal lesion using a redirectable traction control technique (RETRACT), which allows free modification of the traction direction using a clip‐band device (Video 1). A 58‐year‐old man presented with a 40‐mm protruding lesion (type 0‐Is) in the cecum (Figures 1a,b and 2a). First, the mucosal and circumferential incisions were completed with positional changes (Figure 2b,c). A clip‐band traction device was attached to the proximal side of the lesion. The third band was fixed to the opposite normal mucosa, facilitating access to the submucosal layer. However, as dissection progressed, the muscular layer became directly opposed to the dissection plane, making further dissection difficult (Figure 2d–h). Therefore, we detached the third band, grasped the unused second band with a reopenable clip, and redirected the band to achieve the optimal traction direction (RETRACT; Figure 2i–k). This technique enabled access to the remaining submucosal layer and successful en bloc resection (Figure 2l–o). Histopathological examination of the specimen revealed high‐grade dysplasia. In conclusion, RETRACT is a simple technique requiring only clips and one traction‐band device. Even in the narrow cecal space, traction direction can be modified without scope withdrawal, and it may be a useful option in selected cases when the initial traction direction becomes unfavorable.

**FIGURE 1:**
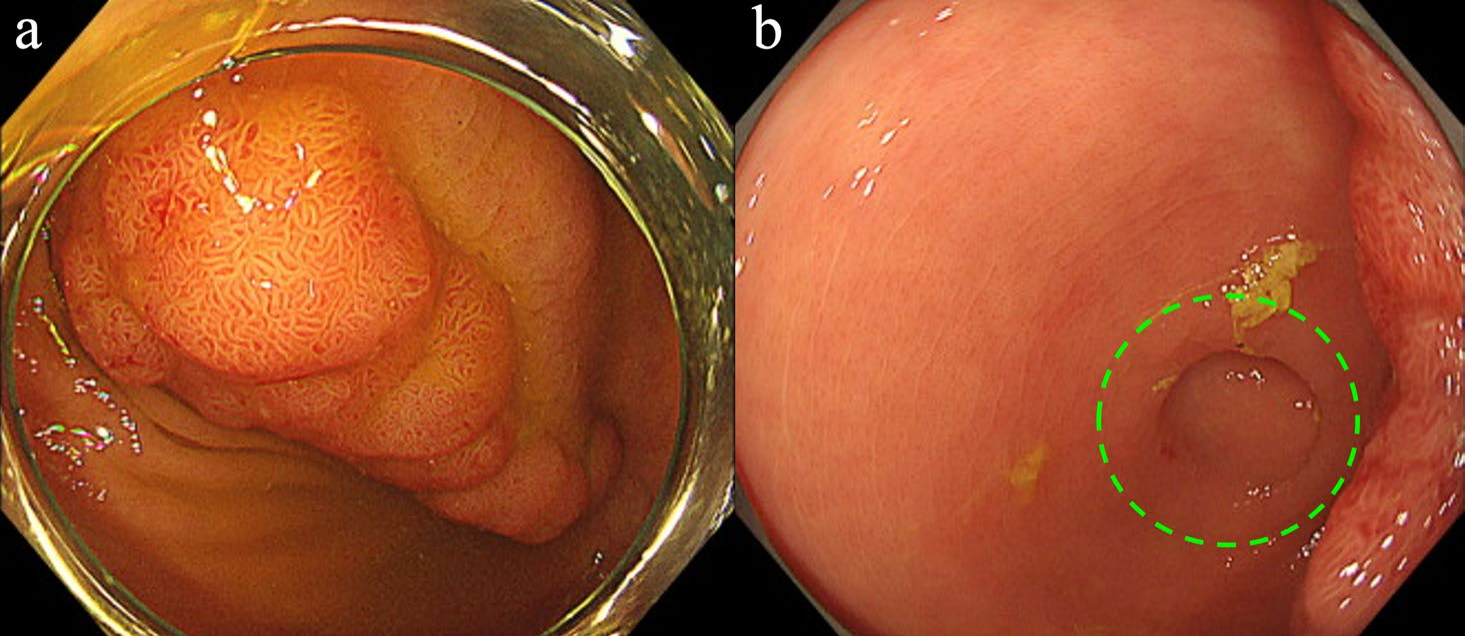
Endoscopic images (a) White‐light image: Lower gastrointestinal endoscopy revealed a protruding lesion (40 mm, type 0‐Is) in the cecum. (b) The lesion was located near the appendiceal orifice (green dotted circle).

**FIGURE 2:**
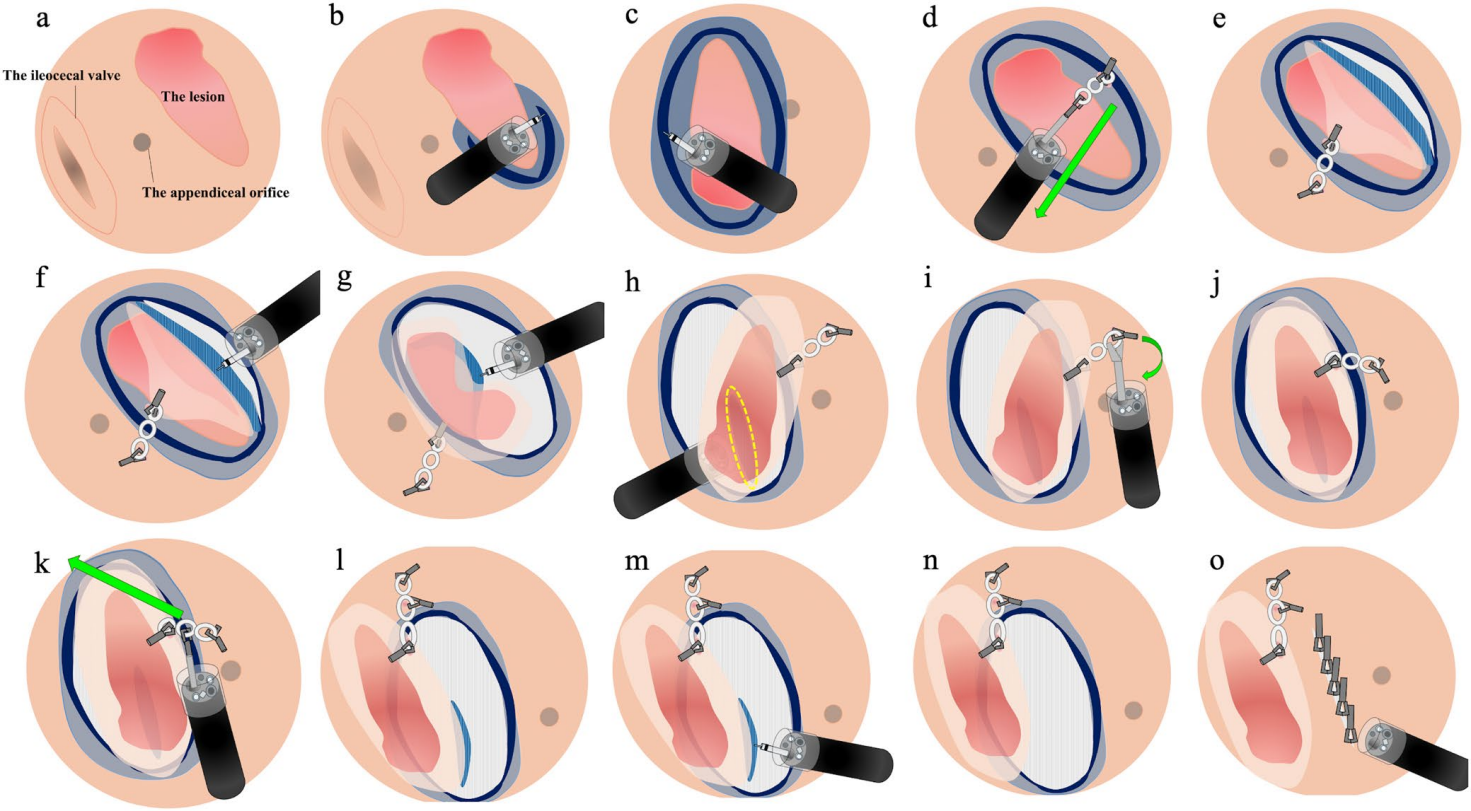
Schematic illustration showing the steps of lesion resection via the redirectable traction control technique (RETRACT) using a clip‐band device. (a) Anatomical relationship among the lesion, appendiceal orifice, and ileocecal valve. (b) Mucosal incision was initiated. (c) A whole circumferential incision was made. (d) The clip‐band traction device was attached to the mucosa on the proximal side of the lesion. After the third band was grasped using the reopenable clip, the band was pulled in the direction of the most effective traction. (e) The third band was clipped to the opposite normal mucosa. (f) This traction facilitated access to the submucosal layer. (g) Submucosal dissection was continued. (h) As submucosal dissection progressed, the muscular layer became elevated (yellow dotted circle), causing difficulty in continuing the dissection. (i) The traction was redirected by detaching the clip anchoring the third band using grasping forceps. (j) The clip securing the third band was detached. (k) The unused second band was grasped with a reopenable clip and redirected to achieve the optimal traction direction; we named this technique the redirectable traction control technique (RETRACT). (l) RETRACT facilitated access to the remaining submucosal layer. (m) Using this technique, we could continue with submucosal dissection. (n) Complete en bloc resection was achieved. (o) The mucosal defect was closed using reopenable clips.

**VIDEO 1:** ESD of the cecal lesion via RETRACT. Video content can be viewed at https://onlinelibrary.wiley.com/doi/10.1111/den.70278.

## Author Contributions

Takahiro Muramatsu wrote and edited the manuscript. Takahiro Muramatsu approved the manuscript. Takahiro Muramatsu, Masakatsu Fukuzawa, and Takao Itoi reviewed the literature and revised the manuscript for intellectual content.

## Funding

The authors have nothing to report.

## Ethics Statement

Informed consent was obtained from the patient for the publication of this report.

## Conflicts of Interest

The authors declare no conflicts of interest.

## Data Availability

The data that support the findings of this study are available from the corresponding author upon reasonable request.

## References

[1] H. Takamaru , Y. Saito , M. Yamada , et al., “Clinical Impact of Endoscopic Clip Closure of Perforations During Endoscopic Submucosal Dissection for Colorectal Tumors,” Gastrointestinal Endoscopy 84 (2016): 494–502. 26774353 10.1016/j.gie.2016.01.014

[2] D. Yang , K. Tao , Q. He , N. Zhang , and H. Xu , “Comparison of the Pocket‐Creation Method With the Conventional Method of Endoscopic Submucosal Dissection for Cecal and Ascending Colon Lesion Resection,” Scandinavian Journal of Gastroenterology 60 (2025): 110–115. 39693389 10.1080/00365521.2024.2440788

[3] H. Ishii , H. Fukuda , Y. Hayashi , et al., “A Traction Wire Facilitates the Pocket‐Creation Method for Endoscopic Submucosal Dissection in the Cecum,” Endoscopy 54 (2022): E776–E777. 35395685 10.1055/a-1807-7571 PMC9735244

[4] H. Takayama , T. Toyonaga , and Y. Kodama , “Endoscopic Submucosal Dissection for a Large Cecal Lesion Using the Circumferential‐Inversion Method,” Digestive Endoscopy 35 (2023): e138–e139. 37608680 10.1111/den.14658

[5] S. Ishizaka , T. Tashima , T. Kawasaki , et al., “Planned Use of a Novel Elastic Traction Device Improves Efficiency in Colorectal Endoscopic Submucosal Dissection: A Propensity‐Score Matched Study,” Surgical Endoscopy 39 (2025): 4641–4651. 40442356 10.1007/s00464-025-11807-0 PMC12222483

